# Metabotropic Glutamate Receptor Blockade Reduces Preservation Damage in Livers from Donors after Cardiac Death

**DOI:** 10.3390/ijms22052234

**Published:** 2021-02-24

**Authors:** Laura Giuseppina Di Pasqua, Clarissa Berardo, Marta Cagna, Roberta Verta, Debora Collotta, Ferdinando Nicoletti, Andrea Ferrigno, Massimo Collino, Mariapia Vairetti

**Affiliations:** 1 Unit of Cellular and Molecular Pharmacology and Toxicology, Department of Internal Medicine and Therapeutics, University of Pavia, 27100 Pavia, Italy; lauragiuseppin.dipasqua01@universitadipavia.it (L.G.D.P.); marta.cagna02@universitadipavia.it (M.C.); mariapia.vairetti@unipv.it (M.V.); 2 Department of Drug Science and Technology, University of Turin, 10125 Turin, Italy; roberta.verta@unito.it (R.V.); debora.collotta@unito.it (D.C.); massimo.collino@unito.it (M.C.); 3 Department of Physiology and Pharmacology, Sapienza University, 00185 Rome, Italy; ferdinando.nicoletti@uniroma1.it; 4 IRCCS Neuromed, 86077 Pozzilli, Italy

**Keywords:** liver, preservation, DCD, MPEP, mGluR5, ischemia/reperfusion

## Abstract

We previously demonstrated that the blockade of mGluR5 by 2-methyl-6(phenylethynyl)pyridine (MPEP) reduces both cold and warm ischemia/reperfusion injury. Here we evaluated whether MPEP reduces the hepatic preservation injury in rat livers from cardiac-death-donors (DCDs). Livers from DCD rats were isolated after an in situ warm ischemia (30 min) and preserved for 22 h at 4 °C with UW solution. Next, 10 mg/Kg MPEP or vehicle were administered 30 min before the portal clamping and added to the UW solution (3 µM). LDH released during washout was quantified. Liver samples were collected for iNOS, eNOS, NO, TNF-α, ICAM-1, caspase-3 and caspase-9 protein expression and nuclear factor-erythroid-2-related factor-2 (Nrf2) gene analysis. Lower LDH levels were detected in control grafts versus DCD groups. An increase in eNOS and NO content occurred after MPEP treatment; iNOS and TNF-α content was unchanged. ICAM-1 expression was reduced in the MPEP-treated livers as well as the levels of caspase-3 and caspase-9. Nrf2, oxidative stress-sensitive gene, was recovered to control value by MPEP. These results suggest that MPEP can be used to reclaim DCD livers subjected to an additional period of cold ischemia during hypothermic storage. MPEP protects against apoptosis and increased eNOS, whose overexpression has been previously demonstrated to be protective in hepatic ischemia/reperfusion damage.

## 1. Introduction

Metabotropic glutamate (mGlu) 5 receptor is a Group I mGlu subfamily of receptors coupled to the inositol trisphosphate/diacylglycerol pathway. Like other mGlu receptor subtypes, the mGlu5 receptor contains a phylogenetically conserved, extracellular orthosteric binding site and a more variable allosteric binding site, located on the heptahelical transmembrane domain [1]. In previous papers, we showed that the mGlu5 receptor is expressed in rat hepatocytes at comparable levels to those observed in the cerebral cortex [2]. In particular, we showed that the mGlu5 receptor has a permissive role in the onset of ischemic injury performed at 37 °C (warm ischemia) in rat and mice hepatocytes; conversely, hepatocytes isolated from livers of mGlu5 receptor knock-out mice are protected against warm ischemic injury [3,4]. Furthermore, using ex vivo mouse models, we recently found that the administration of a negative allosteric modulator of mGluR5, 2-methyl-6(phenylethynyl)pyridine (MPEP), significantly reduced the necrosis in both cold and warm ischemia/reperfusion (I/R) injury [5]. In particular, our data demonstrated the protective role of MPEP in reducing apoptosis using livers submitted to cold storage and reperfusion. The same trend occurred using mice with knock-out of mGluR5 [5]. Although the mGlu5 receptor is considered an interesting pharmacological target in many conditions affecting the CNS and peripheral organs, the liver remains one of the least studied organs with regard to this receptor despite its high expression within this organ.

Liver transplantation remains an effective treatment for patients with end-stage liver disease. Currently, the liver transplant community continues to be challenged by limitations in organ supply, allocation, and quality [6]. Recently, due to marked disparity between the number of patients on the waiting list and the number of donor organs available, there is renewed interest in livers from donors after circulatory death (DCD) [7]. However, patients who receive marginal organs such as DCD livers, have worse prognoses due to an increased incidence of primary non-function (PNF) [8]. Additionally, the incidence of biliary complications and retransplantation increased in the recipients of DCD livers contributing to the high percentage of unutilized DCD livers [9]. Conversely, livers from DCD could be a conspicuous source of organs for transplantation: in various centers, 4–10% of transplants are carried out using DCD livers [10]. The necessity of increasing the quality of organ preservation has made it necessary to consider the use of pharmacological additives to reduce the vulnerability of DCD grafts [11]. Thus, strategies to increase the number of functional donor organs involve pharmacological interventions in DCD procurement techniques that contribute to improved outcomes in DCD transplants [12].

Based on the above reports, we designed a study to test whether the use of MPEP, an mGluR5 allosteric inhibitor, reduces the hepatic preservation injury, increasing the utilization of grafts obtained from DCDs.

## 2. Results

### 2.1. Hepatic eNOS, iNOS, and TNF-Alpha Content after Cold-Storage Preservation

**Figure 1:**
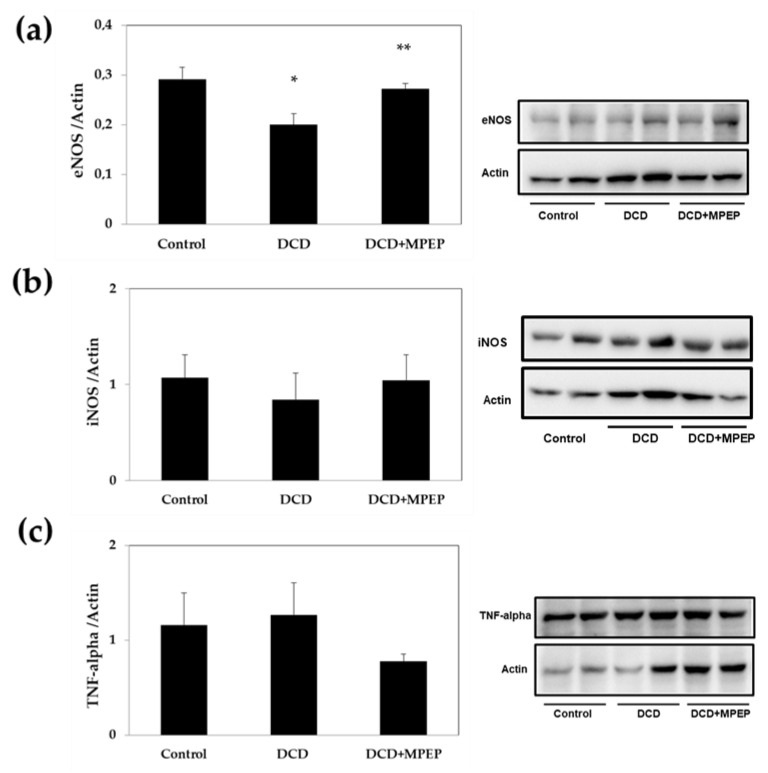
MPEP treatment increases eNOS levels in DCD livers during cold storage. (**a**) eNOS, (**b**) iNOS, and (**c**) TNF-alpha were determined in livers stored for 22 h in UW solution. Control animals underwent cold storage; in the cardiac-death-donor (DCD) group, the livers, after 30 min warm ischemia, were submitted to cold storage. In the DCD-MPEP group, the drug (10 mg/Kg) was administered 30 min before the portal clamping and added to the UW-solution (3 µM). The results are reported as the mean ±SE. * *p* < 0.05 versus control; ** *p* < 0.05 versus DCD.

Since during organ preservation by cold storage, eNOS contributes to increased graft viability [13], we evaluated the effect of MPEP on tissue eNOS content. To simulate the conditions of DCD liver transplantation, rat livers were subjected to 30 min warm ischemia following cardiac arrest and were then preserved by cold storage (22 h). MPEP treatment significantly increased the hepatic eNOS in livers from DCDs to levels comparable to those observed in healthy livers submitted to cold storage (control group) (Figure 1a). Indeed, the evaluation of iNOS levels after cold storage preservation showed comparable content in all groups considered (Figure 1b). The same trend occurred for hepatic TNF-alpha levels (Figure 1c).

**Figure 2:**
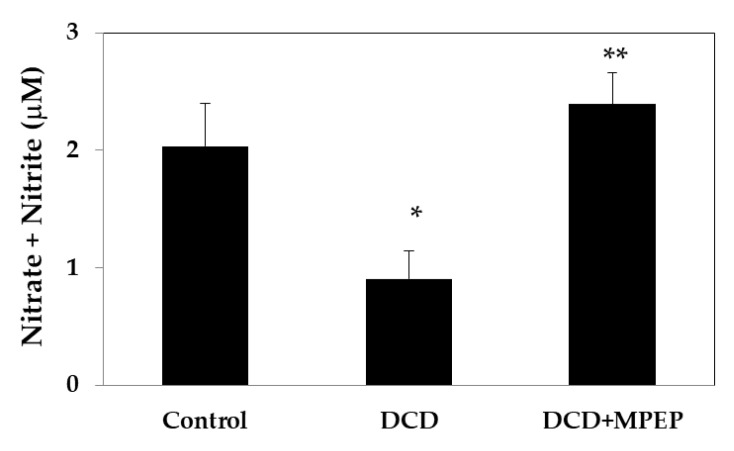
MPEP treatment increases nitrate plus nitrite (NOx) levels in DCD livers during cold storage (22 h in UW solution). Control animals underwent cold storage; in the DCD group, the livers, after 30 min warm ischemia, were submitted to cold storage. In the DCD-MPEP group, the drug (10 mg/Kg) was administered 30 min before the portal clamping and added to the UW-solution (3 µM). The results are reported as the mean ±SE. * *p* < 0.05 versus control; ** *p* < 0.05 versus DCD.

It has been demonstrated that a decrease in NO expression exacerbates I/R injury in human orthotopic liver transplantation [14]. Thus, we evaluated the amount of nitrate plus nitrite (NOx), as an indicator of NO production, after 22 h of cold-storage preservation. A significant decrease in NOx occurred in DCD livers when compared with the control group (Figure 2). MPEP treatment increased the NOx to levels comparable with those found in the control livers.

### 2.2. Effects of MPEP on ICAM-1 and Apoptosis

**Figure 3:**
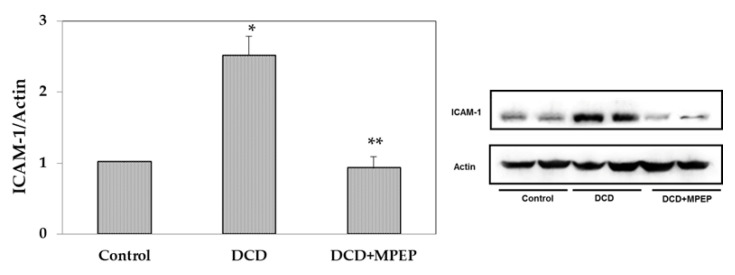
MPEP treatment decreases ICAM-1 in DCD livers during cold storage (22 h in UW solution). Control animals underwent cold storage; in the DCD group, the livers, after 30 min warm ischemia, were submitted to cold storage. In the DCD-MPEP group, the drug (10 mg/Kg) was administered 30 min before the portal clamping and added to the UW solution (3 µM). The results are reported as the mean ± SE. * *p* < 0.05 versus control; ** *p* < 0.05 versus DCD.

Hepatic ICAM-1 expression increases progressively with the cold storage preservation period [15]. In the present study, ICAM-1 levels were evaluated at the end of cold storage, and after 22 h preservation, an increase in ICAM-1 was found in DCD livers compared with those of the control group (Figure 3). Lower ICAM-1 levels, comparable with those found in the control group, were found in DCD livers preserved in presence of MPEP as compared with vehicle-treated group (Figure 3).

**Figure 4:**
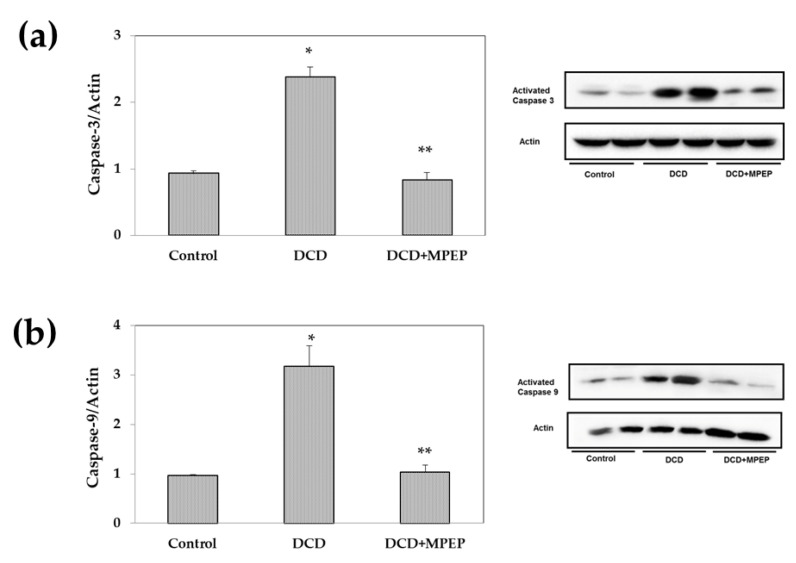
MPEP treatment decreases apoptosis in DCD livers during cold storage. (**a**) Caspase-3 and (**b**) Caspase-9 were determined in livers stored for 22 h in UW solution. Control animals underwent cold storage; in the DCD group, the livers, after 30 min warm ischemia, were submitted to cold storage. In the DCD-MPEP group, the drug (10 mg/Kg) was administered 30 min before the portal clamping and added to the UW solution (3 µM). The results are reported as the mean ±SE. * *p* < 0.05 versus control; ** *p* < 0.05 versus DCD.

Apoptosis is considered a hallmark of cold-induced liver damage [16]. We evaluated the potential role of MPEP in the control of apoptotic death. In cold-storage-preserved livers obtained from DCDs, the blockade of mGlu5 receptor produced a reduction in caspase activation, both caspase-3 and caspase-9, to levels comparable with those found in the control group (Figure 4a,b).

### 2.3. Effects of MPEP on Nrf2 mRNA

**Figure 5:**
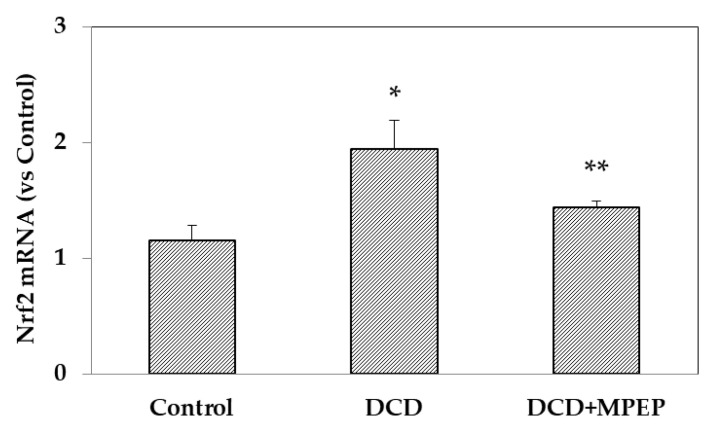
MPEP treatment decreases Nrf2 mRNA in DCD livers during cold storage. Nrf2 was determined in livers stored for 22 h in UW solution. Control animals underwent cold storage; in the DCD group, the livers, after 30 min warm ischemia, were submitted to cold storage. In the DCD-MPEP group, the drug (10 mg/Kg) was administered 30 min before the portal clamping and added to the UW solution (3 µM). The results are reported as the mean ±SE. * *p* < 0.05 versus Control; ** *p* < 0.05 versus DCD.

Nrf2 plays a crucial role as a key oxidative-stress-sensitive gene. Therefore, the present study investigated whether MPEP conferred protection against cold storage in livers obtained from DCD rat via alterations in Nrf2 expression. The results showed that at the end of preservation, an increase in Nrf2 expression in the DCD group compared with the control group occurred; Nrf2 levels were recovered in MPEP-treated DCD group (Figure 5).

### 2.4. Hepatocellular Injury of Livers from DCDs

**Figure 6:**
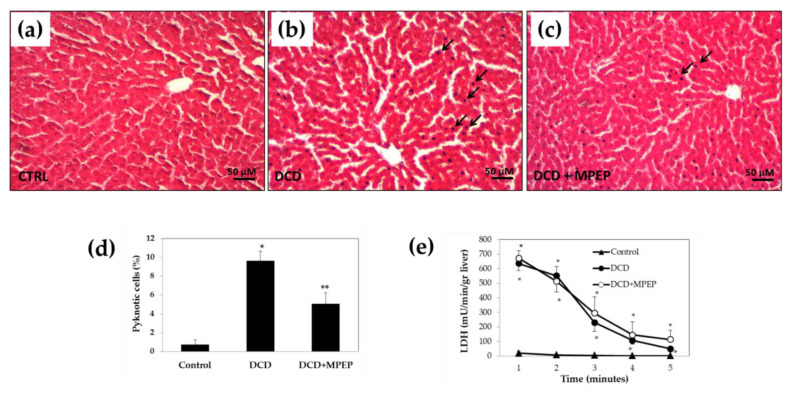
Representative photomicrographs of the morphology (hematoxylin and eosin staining) of DCD livers after cold storage (22 h in UW solution). (**a**) Control animals underwent cold storage; (**b**) in the DCD group, the livers, after 30 min warm ischemia, were submitted to cold storage. (**c**) In the DCD-MPEP group, the drug (10 mg/Kg) was administered 30 min before the portal clamping and added to the UW solution (3 µM). (**d**) Pyknotic cells at the end of cold storage. (**e**) LDH release during washout. Arrows: pyknotic nuclei; scale bar: 50 μm. The results are reported as the mean ±SE. * p < 0.05 versus control; ** *p* < 0.05 versus DCD.

The degree of histological hepatocyte damage differed between control and DCD livers. Livers from control showed well-preserved hepatic architecture (Figure 6a). In DCD livers, an injury to the parenchyma with sinusoid dilatation, areas of nuclear pyknosis and areas of necrotic cells were found (Figure 6b). MPEP did not change the liver morphology, although we found less sinusoid dilatation and pyknotic cells in liver specimens from MPEP-treated rats (Figure 6c,d).

Comparable LDH release in the perfusate during flushing of livers was detected comparing DCD versus MPEP-treated DCD group after 22 h of graft storage (Figure 6e). Lower LDH levels were detected in control grafts versus DCD groups.

## 3. Discussion

Our results demonstrate the ability of MPEP to improve organ quality of DCD livers, most likely mediated by the attenuation of apoptosis and ICAM-1 expression. Furthermore, the protective activity of MPEP appears to result, at least in part, from its capacity to modulate eNOS and NOx content.

Previous results showed that the blockade of mGluR1, which belongs with mGluR5 to Group I mGlu subfamily, attenuates subarachnoid hemorrhage-induced cerebral vasospasm via enhancing eNOS and decreasing active caspase-9, and active caspase-3 [17]. Furthermore, recent results in brain endothelial cells documented a decrease in ICAM-1 mRNA level induced by a systemic lipopolysaccharide administration in mGlu5 receptor knock-out mice [18]. In addition, we previously documented a high concentration of glutamate in isolated rat hepatocytes submitted to warm ischemia [2]. Furthermore, we recently demonstrated the protective effect of MPEP, a mGluR5 blocker, against reperfusion injury in livers submitted to cold storage preservation [5]. Thus we decided to test the efficacy of MPEP in livers from DCDs preserved by cold storage.

In hypoxic–ischemic conditions, as occurs also in organs during cold storage, eNOS promotes vasodilation increasing blood flow, and this event is responsible for increasing graft preservation [19]. Recently, Zhang et al. reported that eNOS-derived NO production significantly attenuates hepatic I/R injury, suggesting that NOS overexpression may constitute a promising therapeutic approach to prevent liver I/R injury following liver transplantation [20]. Russo et al. documented that the addition of simvastatin to the storage solution increased eNOS content, prevented liver damage, and improved endothelial dysfunction [21]. The authors also documented that, during cold storage without treatment, the hepatic endothelial vasoprotective phenotype was rapidly lost, and the hepatic expression of eNOS was significantly reduced already after 1 h of cold storage. In the present study, we also documented a decrease in eNOS and NOx after cold storage of DCD livers; furthermore, we reported the protective effect of MPEP able to increase eNOS and NOx during preservation. Comparable levels of NOx were previously reported in livers after cold ischemia injury [22]. No significant differences in iNOS content were found. In control animals, during reperfusion after cold storage, we previously documented an iNOS lower levels and no significant difference in eNOS expression in livers from MPEP treated rats. An explanation of these results is related to different experimental conditions: in the present study MPEP treatment was evaluated in DCD livers during cold storage and likely the mGlu5 receptor blockade is associated with vasodilation and, therefore, an increased blood flow during ischemia. On the contrary, iNOS activation needs more time to be activated by different stimuli as pro-inflammatory cytokines and here we reported no changes in TNF-alpha during cold storage. Our results are in line with previously reported investigations showing how the addition of melatonin to cold storage solution increased eNOS expression but not iNOS activation [23]. In addition, genetic overexpression of eNOS attenuates hepatic I/R injury, and a deficiency of eNOS has been shown to exacerbate injury in hepatic models of I/R [24].

The pathogenic events occurring during I/R trigger a series of deleterious effects that include induction of apoptosis in hepatocytes and increased expression of adhesion molecules, which together lead to massive tissue destruction [25]. To ameliorate the severity of liver I/R, several therapeutic strategies are currently being pursued, including the inhibition of apoptosis [26]. Of interest already within the context of ischemia, the hypoxic environment initiates the expression of several genes involved in apoptosis [27]. Using a rat model of cold storage and reperfusion, we already demonstrated the MPEP capacity to prevent apoptosis activation [5]. The present data show the ability of MPEP to reduce caspase activation also in DCD livers during cold storage preservation. The same trend was found, for the first time, for ICAM-1 content. ICAM-1 serves as an indicator for endothelial cell activation, and its expression increased progressively with cold preservation [15]. In particular, when cold ischemia was extended to 16 and 24 h, ICAM-1 expression showed significant upregulation [15]. Furthermore, both ICAM-1 expression and apoptotic cell death have been pharmacological modulated to attenuate post-ischemic liver injury after prolonged cold storage [28].

The pathogenesis of liver cold storage injury also involves the formation of reactive oxygen species (ROS) that have been shown to influence the expression of key oxidative-stress-sensitive genes. One of the most relevant transcription factors that is upregulated in response to oxidative stress is nuclear factor (erythroid-derived 2)-like 2 (Nrf2) [29]. We found an increase in Nrf2 expression in DCD liver and a significant reduction to control levels in DCD livers treated with MPEP. Recently Xue et al. reported that, after reperfusion, cold-storage livers from DCD exhibit a reduction in Nrf2 mRNA [30]. This discrepancy with our data is probably due to the different samples used: we collected the liver biopsies at the end of the preservation period without reperfusion. Recently, no difference in Nrf2 mRNA at the end of the cold storage of porcine DCD kidney vs. control healthy kidney was found [31]. We supposed that during liver cold storage of DCD livers Nrf2 activation could be prevented by MPEP administration.

It is accepted that the initial factor determining poor graft microcirculation during cold preservation is the hepatic endothelium damage associated with persistent vasoconstriction, upregulation of adhesion molecules, and oxidative stress. Of note, these events reduce the blood-derived biomechanical stimuli, which have been demonstrated to be protective [32]. Considering the intimate cellular crosstalk between endothelium cells and hepatocytes, it is very possible that the injury of endothelium cells due to cold storage may negatively affect hepatocyte viability and vice versa [32]. It might be that MPEP was able to counteract some of these events thus protecting DCD livers from cold preservation injury.

The pharmacological interventions represent a chance to reduce preservation injury, especially in particularly vulnerable livers such as those from DCDs. The use of DCDs for liver transplantation could be made possible by treatment for reducing the effects of both warm and cold ischemia; the modulation of mGlu5 receptor by MPEP might provide a new approach for the successful use of marginal livers such as those obtained from DCDs.

Since the current work investigated only the effect of mGluR5 blockade in DCD livers during cold preservation, further studies need to evaluate MPEP-induced protection after warm reperfusion as well as in rat orthotopic liver transplantation models. Although these are preliminary results, they represent the basis for further investigations to clarify the biological and clinical significance of MPEP: its potential clinical implications and preventive use could be considered for the preservation of DCD grafts.

In conclusion, the shortage of liver grafts due to the increase in the number of patients requiring liver transplantation suggests the use of DCDs as an additional source that would increase the donor pool. MPEP increases eNOS and protects DCD livers against apoptosis. The mGluR5 modulation represents a novel pharmacological approach for marginal liver preservation such as those from DCDs. Future experimental investigations in a liver transplantation model are needed to put these results into perspective.

## 4. Materials and Methods

### 4.1. Chemicals

2-methyl-6(phenylethynyl)pyridine (MPEP) hydrochloride was purchased from Tocris Bioscience (Bio-techne SRL, Milan, Italy). *N*-(2-hydroxyethyl)-piperazine-*N*′-(2-ethanesulphonic acid) (HEPES) and all chemicals were purchased from Merck (Milan, Italy).

### 4.2. Animals and Surgery

Male Wistar rats (Harlan-Nossan, Italy), weighing 250–300 g, were allowed free access to water and food until the beginning of all experiments. The use and care of animals in this experimental study were approved by the Italian Ministry of Health and by the University Commission for Animal Care (Protocol number: 3.13.2010). Rats were anesthetized with sodium pentobarbital (40 mg/kg intraperitoneally). After median laparotomy followed by bilateral subcostal incisions, the animals received 500 units of heparin via the inferior vena cava (5000 IU/mL, Marvecs Services, Agrate Brianza—MI) in accordance with the experimental method reported in the literature [33]. The use of heparin regards the organs obtained from the DCD as reported in clinical studies [34]. After 2 min, a phrenotomy was performed to sacrifice the animal. The warm ischemic time started after cessation of blood flow to the liver. During warm ischemia, the portal vein was cannulated with a 16 G catheter (Johnson & Johnson, Arlington, UK). After 30 min of warm ischemia, the liver was washed out with 50 mL Ringer Lactate via the portal vein cannula. The washout solution drained away by cutting the infrahepatic caval vein. The suprahepatic caval vein was then excised, the infrahepatic caval vein was ligated, and the liver was removed. Then, livers were preserved for 22 h by cold storage using UW solution. In a group, of livers the MPEP was administered 30 min before warm ischemia (10 mg/kg, dissolved in saline solution and 10% ethanol) and added to the UW solution (3 µM) (*n* = 5). The MPEP concentrations have been previously tested [3]. In the control DCD group, saline solution added with 10% ethanol was both administered as a vehicle 30 min before the portal clamping and added to the UW solution (*n* = 5). Livers from heart-beating donor animal (Control) (*n* = 4) followed the same procedure of DCD without 30 min ischemia. In order to comply with the principle of the three Rs (Replacement, Reduction, Refinement) for care and use of animals, attention was paid to (i) standardizing the procedures, performed by the same operator to avoid operator variability, (ii) minimizing the animal suffering, (iii) minimizing the number of animals. After the isolation, the livers were flushed in situ with ice-cold UW for 2 min and maintained at 4 °C in this solution for 22 h. After cold storage, the livers were washed with Ringer lactate solution (20 mL), and the samples were taken from the flush [35]; liver samples were quickly removed and frozen in liquid nitrogen.

Hepatocyte viability was assessed by release, into the effluent washout perfusate, of lactate dehydrogenase (LDH) that was evaluated as previously described [36].

Liver concentrations of nitrate plus nitrite (NOx) were determined using a colorimetric assay kit (Cayman Chemical Company, Ann Arbor, MI, USA, No. 780001) according to the manufacturer’s instructions.

### 4.3. Western Blot Assay

Liver tissue samples were homogenized in an ice-cold CelLytic Buffer supplemented with Protease Inhibitor Cocktail and centrifuged at 15,000× *g* for 10 min. The collected supernatant was divided into aliquots containing the same amount of proteins and stocked at −80 °C. Samples of liver extracts containing the same amount of proteins were separated in SDS-PAGE on 7.5% acrylamide gels and transferred to PVDF membrane. Unspecific sites were blocked for 2 h with 5% Bovine Serum Albumin (BSA) in TBS Tween (20 mM Tris/HCl, 500 mM NaCl, pH 7.5, 0.1% Tween 20) at 4 °C. The membranes were incubated with primary antibodies overnight at 4 °C, under gentle agitation. Primary antibodies against rabbit polyclonal anti-Actin were used at 1:5000 dilution; rabbit polyclonal anti-eNOS, anti-iNOS, anti-ICAM-1, anti-Caspase-3, and anti-Caspase-9 were used at 1:1000 dilution; goat polyclonal antibody anti-TNF-alpha was used at 1:100 dilution. Membranes were washed in PBS Tween (Na_2_HPO_4_ 8 mM, NaH_2_PO_4_-H_2_O 2 mM, NaCl 140 mM, pH 7.4, 0.1% Tween 20) and incubated with peroxidase-conjugated secondary anti-Rabbit or anti-Mouse antibodies at a 1:2000 dilution. Anti-iNOS was purchased from Cayman Chemical (Ann Arbor, MI, USA), eNOS, ICAM-1, caspase 3. Caspase 9 and TNF-alpha were bought from Santa Cruz Biotechnology. Immunostaining was revealed with BIO-RAD Chemidoc XRS+ visualized using the ECL Clarity BIO-RAD (Milan, Italy). Bands intensity quantification was performed by BIO-RAD Image LabSoftware™ 6.0.1 (Milan, Italy).

### 4.4. RT-PCR

Total RNA was isolated from the median lobe of frozen livers with TRI reagent (Sigma-Aldrich, St. Louis, MO, USA) following the Chomczynski method [37]. RNA was quantified by measuring the absorbance at 260 nm, and purification was assessed using the ratio between 260/280 nm with T92+ UV Spectrophotometer. The cDNA was generated using iScript Supermix (Bio-Rad, Milan, Italy) [38]. The qPCR reactions were performed by CFX96TM Real-Time System (Bio-Rad, Milan, Italy) using 10 μL of SsoAdvancedTM SYBR^®^ Green Supermix (Bio-Rad, Milan, Italy), 1 μL of the oligonucleotide primer (10 pmol/μL), and 2 μL of cDNA (2.5 ng/μL) to reach a final volume of 20 μL/well. Nrf2, GAPDH, USP28, and HPRT-1 gene amplification efficiencies were established by means of calibration curves (102%, 104%, 104%, 99.3%, respectively). The expression of the reference genes remained constant in the considered experimental groups. The amplicon context sequence of the primers (Bio-Rad, Milan, Italy) Nrf2 (Unique assay ID: qRnoCID0008218), GAPDH (Unique assay ID: qRnoCID0057018), USP28 (Unique assay ID: qRnoCID0015123), and HPRT-1 (Unique assay ID: qRnoCED0057020) are reported in Table 1, which displays the amplicon sequences with additional base pairs added to the beginning and/or end of the sequence. This is in accordance with the minimum information for the publication of real-time quantitative PCR experiments (MIQE) guidelines, as reported by Bustin et al. [39]. The results were normalized to the endogenous controls, and the fold change of the gene expression was calculated using threshold cycle (Ct) values.

### 4.5. Histological Analysis

At the end of cold storage preservation, liver biopsies were rapidly removed, fixed in 2% p-formaldehyde in 0.1 M phosphate buffer at pH 7.4 for 24 h and processed routinely until they were embedded in Paraplast wax. Sections were cut at 7 μm and stained with Hematoxylin and Eosin (H & E) for histological examination. The samples were photographed and analyzed using a microscope equipped with a digital camera and (Nikon 800). Histological assessment included the measurement of the percentage (average of four counts) of cells with pyknosis.

### 4.6. Statistical Analysis

Statistical Analysis was performed using MedCalc Statistical Software version 18.11.3 (MedCalc Software bvba, Ostend, Belgium; https://www.medcalc.org; 2019). Statistical analysis was performed with one-way ANOVA with Tukey’s multiple comparison test, as post-hoc test or Kruskal–Wallis and Dunn’s test, as appropriate. To assess normality of variance changes Kolmogorov–Shapiro normality test was used. Results are expressed as mean value ± standard error (SE). The value of *p* < 0.05 was considered the criterion for statistical significance.

## Figures and Tables

**Table 1:** Amplicon sequences of primers used in this study. Nuclear factor (erythroid-derived 2)-like 2 (Nrf2 or Nfe2l2) was the gene of interest to be analysed. Glyceraldehyde-3-Phosphate Dehydrogenase (GAPDH), Ubiquitin Specific Peptidase 28 (USP28), and Hypoxantine phosphoribosyltransferase-1 (HPRT-1) were used as reference genes.

Gene	Amplicon Sequence
Nrf2 (Nfe2l2)	TACCCCAAGATCTATGTCTTCCTCCAAAGGATGTCAATCAAATCCAT GTCCTGCTGGGACTGTAGTCCTGGCGGTGGAATTCCAAGTCCATCAT GCTGAGGGCGGACGCTGCGCTAGGGC
GAPDH	TGATGGCAACAATGTCCACTTTGTCACAAGAGAAGGCAGCCCTGGA ACCAGGCGTCCGATACGGCCAAATCCGTTCACACCGACTTCACCATCTTGTCTATGAGACGAGGCTGGCACTGCACAAGAATGCGGCTGTCTCTA
USP28	CCGAGACGGGTCTGAAGCAGGGCTTATTAAGGCATTTCATGAAGAGTACTCCAGGCTCTATCAGCTTGCCAAAGAGACACCCACCTCTCACAGTGATCC
HPRT-1	TTCATGCAAAAGCTTTACTAAGTAGATGGCCACAGGACTAGAACGT CTGCTAGTTCTTTACTGGCCACATCAACAGGACTCTTGTAGATTCAAC TTGCCGCTGTCTTTT

## Data Availability

The data presented in this study are available on request from the corresponding author.
